# Cerebrospinal fluid adenosine deaminase activity: A complimentary tool in the early diagnosis of tuberculous meningitis

**DOI:** 10.1186/1743-8454-3-5

**Published:** 2006-03-30

**Authors:** Rajpal S Kashyap, Rani P Kainthla, Anju V Mudaliar, Hemant J Purohit, Girdhar M Taori, Hatim F Daginawala

**Affiliations:** 1 Biochemistry Research Laboratory, Central India Institute of Medical Sciences, 88/2 Bajaj Nagar, Nagpur-440010, India; 2 Environmental Genomics Unit, NEERI, Nehru Marg, Nagpur-440020, India

## Abstract

**Background:**

Tuberculous meningitis (TBM) is the commonest form of neurotuberculosis caused by *Mycobacterium tuberculosis *bacilli (MTB). The diagnosis of TBM is often difficult. A reliable, cost-effective and rapid diagnostic test, which can be performed in any standard pathology laboratory, could be of help in the diagnosis of TBM. In the present study we measured the adenosine deaminase (ADA) activity in cerebrospinal fluid (CSF) of TBM and non-TBM patients.

**Method:**

ADA activity in CSF was determined according to a method based on the Berthlot reaction, which is the formation of a colored indophenol complex from ammonia liberated from adenosine, and quantified spectrophotometrically.

**Results:**

The CSF ADA activity from TBM patients was compared with CSF ADA from non-TBM infectious meningitis patients, and from patients with non-infectious neurological disorders. The mean CSF ADA activity was found to be significantly higher in CSF of TBM patients, 14.31 ± 3.87 (2.99–26.94), mean ± SD with range, than in the CSF from non-TBM infectious meningitis, 9.25 ± 2.14 (4.99–13.96) and from the non-infectious neurological disorders group, 2.71 ± 1.96 (0.00–7.68), *P *< 0.0001 for both comparisons. A cut-off value of 11.39 U/L/min for the TBM patients was calculated from the mean + SD of the non-TBM patients. The ADA test gave a sensitivity of 82% and a specificity of 83% for infectious TBM when this cut-off value was used.

**Conclusion:**

This study demonstrated that ADA activity in the CSF of TBM patients, using a cut-off value 11.39 U/L/min, can be useful for the early differential diagnosis of TBM. This test can be performed in any pathology laboratory where more sophisticated methods are not available.

## Background

Tuberculous meningitis (TBM) is the infection of the meninges caused by *Mycobacterium tuberculosis *(MTB). The diagnosis of TBM is complicated as it causes various clinical manifestations, which overlap with those of other chronic diseases of the central nervous system (CNS) such as viral and pyogenic meningitis [1]. The initiation of anti-TB medication in suspected TBM patients can often be delayed because of a lack of confidence in the presently available laboratory tests [2,3]. Most of the tests developed for the early diagnosis of TBM are not sensitive [4] and although some other tests are useful, they may not be affordable for routine use [5,6]. A reliable and rapid diagnostic test, which can be performed in any standard pathology laboratory, could be of help in diagnosis of TBM.

Adenosine deaminase (ADA) is an enzyme that catalyzes the deamination of adenosine, forming inosine in the process [7]. The chief physiological function of ADA is related to lymphocytic proliferation and differentiation [8]. As a marker of cellular immunity, activity is found to be elevated in those diseases in which there is a cell-mediated immune response [9]. Numerous previous studies have demonstrated that CSF-ADA estimation is useful in the diagnosis of TBM and can differentiate TBM from normal subjects or from patients with other neurological disorders [10-12]. However, the results were variable and one study has shown that ADA is of limited value as it was also raised in other types of meningitis especially pyogenic meningitis [13]. In this paper we describe a prospective study to evaluate the reliability of ADA activity in the CSF for diagnosis in patients with TBM, patients with non-TBM infectious meningitis and patients with non-infectious neurological disease. A cut-off value for CSF ADA activity has been calculated for the diagnosis of TBM.

## Materials and methods

### Patients

A total of 281 patients (TBM = 117, pyogenic meningitis = 41, viral meningitis = 19, and 104 control subjects with non-infectious neurological disorders) were included in the present study. Patients included in the study were those admitted to the Neurology Department of Central India Institute of Medical sciences (CIIMS) between 1^st ^January 2003 and the end of February 2004. All patients were above the age of 20 years. CSF samples for ADA estimations and other tests were obtained before starting any treatment in all cases of neurological disorders including viral, bacterial, and mycobacterial meningitis. The Institutional Ethics Committee of Central India Institute of Medical Sciences, Nagpur, approved the study.

### Patient Groups (Table 1)

**Table 1:** The mean ADA activity (with range) in the CSF of TBM patients (n = 117), non-TBM infectious meningitis patients (n = 60) and control patients with non-infectious neurological disorders (n = 104). The data are expressed as mean ± SD

**Patient groups**	**ADA activity (U/L/min) Mean ± SD**	**Range**
**1. Tuberculous Meningitis (n = 117)**	**14.31 ± 3.87**	2.99–26.94
Culture positive (n = 27)	17.67 ± 4.18	9.01–26.94
Clinically suspected (n = 90)	13.29 ± 3.16	2.99–21.02
**2. Non TBM infectious meningitis (n = 60)**	**9.25 ± 2.14**	4.99–13.96
Pyogenic meningitis (n = 41)	10.11 ± 1.99	5.11–13.96
Viral meningitis (n = 19)	7.39 ± 0.93	4.99–9.00
**3. Non-infectious neurological disorders (n = 104)**	**2.71 ± 1.96**	0.00–7.68
Headache (n = 32)	0.98 ± 0.19	0.11–1.20
Stroke (n = 29)	4.18 ± 1.19	1.92–5.83
Venous sinus thrombosis (n = 13)	1.82–4.12	
Guillian-Barré syndrome (n = 12)	5.38 ± 2.16	2.63–7.68
Epilepsy (n = 6)	2.36 ± 0.79	1.01–3.18
Other neurological disorders (n = 12)	1.18 ± 0.47	0.5–1.87

#### 1. Tuberculosis Meningitis patients (n = 117)

A: Clinically confirmed cases (n = 27): Confirmed by the presence of Mycobacterium tuberculosis in CSF by staining and/or culture.

B: Clinically suspected patients (n = 90): This group had negative cultures with all of the following observations:

a: Sub-acute or chronic fever with features of meningeal irritation such as headache, neck stiffness and vomiting, with or without other features of CNS involvement.

b: CSF samples showing raised protein levels, and/or decreased glucose (CSF: blood glucose ratio <0.5), and/or pleocytosis with lymphocytic predominance.

c: Good clinical response to antituberculous drugs.

#### 2. Non-TBM infectious pyogenic and viral meningitis patients (n = 60)

A: Pyogenic meningitis (n = 41): Confirmed cases (n = 9): Presence of pathogenic bacteria in CSF by staining and/or culture.

Clinically suspected (n = 32): This group included the culture negative cases with all of the following observations:

a: Fever and/or signs of meningeal irritation (patients who have undergone cranial surgery to treat tumor(s), stroke, or head injury and who have received antibiotics), or high fever and/or signs of meningeal irritation with or without CNS manifestations (patients who received broad-spectrum antibiotics).

b: CSF findings showing increased proteins, decreased glucose (CSF: blood glucose ratio <0.2), and/or pleocytosis with a predominance of polymorphonuclear cells.

c: Good clinical response to broad-spectrum antibiotics.

B: Viral meningitis patients (n = 19): This group includes suspected patients with the following observations:

a: Acute onset of fever and symptoms and signs of meningeal irritation.

b: CSF samples showing mild increase in protein, glucose often normal and pleocytosis, predominantly lymphocytic.

c: No clinical evidence for extra cranial tuberculosis.

#### 3. Non-infectious neurological disorders group (n = 104)

All other patients who had no evidence of CNS or extra CNS bacterial or viral infections were grouped in the non-infectious/control group. Patients included in this group had chronic intractable headache, status epilepticus, stroke and others disorders (Table 1).

### Sample collection

CSF samples were collected by standard lumbar puncture. Approximately 3 ml of CSF was obtained; 2 ml of CSF was used for total and differential cell count, biochemistry, and smear for Gram's, India ink, and acid fast bacilli (AFB) staining and the remaining CSF was used for ADA estimation. All the samples were stored at 4°C until further analysis.

### ADA activity measurement

ADA activity in CSF was determined at 37°C according to the method of Guisti and Galanti [14] based on the Berthlot reaction, that is the formation of colored indophenol complex from ammonia liberated from adenosine and quantified spectrphotometrically (U.V.Visible spectrophotometer.Remi.Model C-24). One unit of ADA is defined as the amount of enzyme required to release 1 mmol of ammonia/min from adenosine at standard assay conditions. Results were expressed as units per litre per minute (U/L/min). The assays were performed in triplicate and blind to the diagnosis.

### Data analysis

Results are expressed as mean ± SD with Range. To compare the mean ADA activity between the TBM, non-TBM infectious meningitis and non-infectious neurological disorders groups, the Kruskal Wallis test (non-parametric ANOVA) with the Dunnett post-test was used. A *P *value less than 0.05 was considered significant. A cut off value of ADA activity for TBM patients was calculated from the mean plus SD of ADA activity in the non-TBM infectious meningitis group. The sensitivity (true positive rate) for the test was calculated as: [the number of samples in the TBM group with ADA activity ≥ (mean+SD) of ADA activity in the non-TBM infectious meningitis group divided by the total number of samples in TBM group] × 100. The specificity (true negative rate) for the test was calculated as: [the number of samples in non-TBM infectious meningitis group with ADA activity < (mean+SD) of ADA activity in the non-TBM infectious meningitis group divided by the total number of samples in non-TBM infectious meningitis group] × 100.

## Results

The total number of patients studied was 281. Of these, 27 were confirmed for TBM, 90 were suspected for TBM, 60 had non-TBM infectious meningitis (pyogenic meningitis, 41, and viral meningitis, 19) and 104 had other CNS disorders.

**Figure 1:**
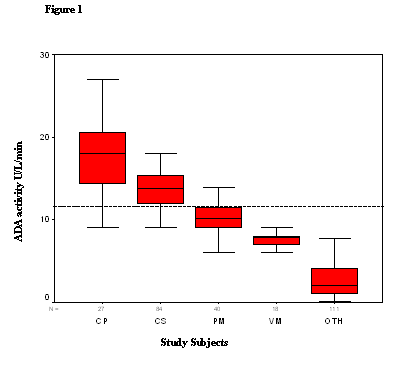
Box plots of CSF ADA activity in TBM (CP-culture positive; CS-clinically suspected), non-TBM infectious meningitis (PM- pyogenic meningitis; VM-viral meningitis) and control group of non-infectious neurological disorders (OTH). The plots show the 90^th ^percentile (bars), 75^th ^and 25^th ^percentile (box) and median (bar in box). N = numbers of individuals in each group. Dashed line represents the calculated ADA cut off value.

Table 1 depicts the mean ADA activity in the CSF of different patient groups. The mean ADA activity in TBM patients was 14.31 ± 3.87 (2.99–26.94), significantly higher than the non-TBM infectious meningitis group, 9.25 ± 2.14 (4.99–13.96); *P *< 0.0001, and also higher than the non-infectious neurological disorders group, 2.71 ± 1.96 (0.00–7.68); *P *< 0.0001. A significant difference in the mean ADA activity was noted also in the CSF of culture-positive TBM patients, 17.67 ± 4.18 (9.01–26.94), when compared with the clinically suspected TBM patients, 13.29 ± 3.16 (2.99–21.02); *P *< 0.0001. The cut off value of the ADA activity for the diagnosis of TBM patients was, calculated as 9.25 + 2.14 = 11.39 U/L/min. Box plots of the ADA activity in the CSF of culture positive and clinically suspected TBM patients, non-TBM infectious meningitis and non-infectious neurological disorders groups are shown in Figure 1, together with the 90^th ^percentile range, 75^th ^and 25^th ^percentiles.

Table 2 shows the number and percentage of patients in each group that fall above or below the cut-off value. Sensitivity in the TBM group was 96% (96% of the culture positive samples were above the cut-off value) whereas it was only 78% in the clinically suspected group. In the non-TBM infectious meningitis group, 24% in the pyogenic meningitis group were above the cut-off value, but none of the viral meningitis samples were over the cut-off value. For the non-infectious neurological disorder group, none of the patients had ADA activity above the cut-off value. The sensitivity of the ADA test to differentiate between TBM and non-infectious meningitis was 82% and the specificity was 83% when the cut-off value of 11.39 U/L/min was used. The 95% confidence interval for sensitivity was 90.19 (upper limit) and 73.91 (lower limit) while the 95% confidence interval for specifity was 88.99 (upper limit) and 77.67 (lower limit).

**Table 2:** The number and percentage of patients positive and negative for the CSF ADA test using the diagnostic cut-off value of > 11.39 U/L/min for the TBM patients, the non-TBM infectious meningitis patients and the control group with non-infectious neurological disorders. The sensitivity of the test for positive diagnosis was 82% and the specificity was 83%. The 95% confidence interval for sensitivity is between 73.91 and 90.19, while 95% confidence interval for specificity is between 77.67 and 88.99.

Patient groups	Number of patients with ADA activity >11.39 U/L/min	Number of patients with ADA activity < 11.39 U/L/min
**1. Tuberculous Meningitis (n = 117)**	**96 (82%)**	**21 (18%)**
Culture positive (n = 27)	26 (96%)	1 (4%)
Clinically suspected (n = 90)	70 (78%)	20 (22%)
**2. Non-TBM infectious meningitis (n = 60)**	**10 (17%)**	**50 (83%)**
Pyogenic meningitis (n = 41)	10 (24%)	31 (76%)
Viral meningitis (n = 19)	0 (0%)	19 (100%)
**3. Non-infectious neurological disorders (n = 104)**	**0**	**104 (100%)**
Headache (n = 32)	0	32 (100%)
Stroke (n = 29)	0	29 (100%)
Venous sinus thrombosis (n = 13)	0	13 (100%)
Guillian-Barré syndrome (n = 12)	0	12 (100%)
Epilepsy (06)	0	06 (100%)
Other neurological disorders (n = 12)	0	12 (100%)

## Discussion

TBM remains a major global health problem and even in developed countries there is a resurgence of tuberculous infection due to the growing number of people infected with human immunodeficiency virus (HIV) [15,16]. Early confirmatory diagnosis of TBM is difficult to establish because of its pleomorphic clinical presentation [3,17]. Delayed diagnosis and treatment may be associated with many serious CNS complications [18]. The most commonly used laboratory method for the definitive diagnosis of TBM is to demonstrate the presence of tubercle bacilli either by smear and/or culture. However, direct smear methods are often negative in CSF samples and culturing of MTB takes 4–6 weeks to show the growth [19,20]. Newer methods such as those involving the amplication of bacterial DNA by the PCR and comparable systems, are incompletely assessed and not available for widespread use in the developing countries. The sensitivity of the PCR technique varies from 33% to 90% and the specificity from 88% to 100% [21]. Various immunoassays such as antigen and/or antibody detection in CSF samples have been developed with variable sensitivities and specificities [2,9,10,22-24]. Hence, despite extensive work on TBM, only few diagnostic tests are available. Recently we have reported on a 30 kD protein antigen comprising of antigen 85 complex in CSF of TBM patients by indirect ELISA method with a sensitivity of 89% and specificity of 97% [25].

ADA has been considered as a marker of cell-mediated immunity and its activity has been observed in various infections including TBM [9]. Considering that both humoral and cell-mediated immunity play an important role in TBM infection, it has been suggested that ADA activity in CSF may help differentiate TBM from non-TBM infectious meningitis and non-infectious neurological disorders, and this has been discussed earlier by various workers [11,12,26].

In the present study we have calculated an ADA cut-off value of 11.39 U/L/min in CSF for the diagnosis of TBM infection. Using this we have demonstrated a sensitivity of 96% and 78% in the CSF of culture-positive and clinically-diagnosed TBM patients, respectively. False positive results were noted in 24% of pyogenic meningitis cases. No other control group gave false positive results.

Earlier, various workers have reported the reliability of CSF ADA activity in TBM patients using different cut off values. Pettersson et al. reported sensitivity of 100% and specificity of 99% when a cut off value of 20 U/L/min was used, but in that study there were only three enrolled tuberculous meningitis patients [12]. Baro et al proposed a cut off value of 6.5 U/L/min and showed sensitivity of 83.3% and specificity of 85.3% [11]. However, this study used only 12 cases of TBM patients, and pyogenic and viral meningitis were not distinguished from the group with other central nervous system diseases. Similarly increased CSF ADA levels have been reported in childhood TBM with adverse neurological outcome [27]. However, Gambhir et al reported a low sensitivity of 44% and specificity of 75% for ADA test with a cut off value 8 IU/L/min, which showed overlap between TBM and non-TBM patients, especially for infectious neurological disorders like pyogenic meningitis [28].

The laboratory method for measuring ADA is inexpensive, relatively simple to perform and can also be adapted to an autoanalyser. It may thus be useful in laboratories with limited resources, especially in underdeveloped and developing countries like India where the incidence of tuberculosis is very high and where, despite extensive work on pulmonary and extra pulmonary tuberculosis, only a few diagnostic kits are available for tuberculous meningitis.

## Conclusion

In conclusion, the determination of ADA activity in CSF of TBM patients using cut off value of 11.39 U/L/min can be useful for the early differential diagnosis of TBM and it is cost effective. However CSF ADA should be interpreted judiciously in the light of the patient's clinical condition and CSF characteristics since there is a wide range of CSF ADA activity in TBM. In addition, there is overlap with ADA activity in some pyogenic meningitis patients. Overall our study suggests that measurement of ADA activity in CSF of TBM patients with a cut off value of 11.39 U/L/min can be useful as a supporting test for the differential diagnosis.

## Declaration of competing interests

The author(s) declare that they have no competing interests.

## Authors' contributions

RSK carried out the study design, data collection, statistical analysis, data interpretation, literature search, and manuscript preparation; RPK and AVM assisted in data analysis collection; HJP participated in the preparation of the manuscript, data interpretation, and study design; GMT provided assistance in preparation of the manuscript, data interpretation, study design, and funds collection; and HFD supervised the study design, statistical analysis, data interpretation, manuscript preparation, and literature search. All authors have read and approved the final version of the manuscript.
